# 基于血清与脑脊液脂质代谢组学的新生儿败血症生物标志物分析

**DOI:** 10.3724/SP.J.1123.2025.06003

**Published:** 2026-03-08

**Authors:** Weixiang WU, Fuqiang DIAO, Junfei GUO, Chunming GU, Lihong WU, Mingyong LUO

**Affiliations:** 南方科技大学附属妇女儿童医院临床检验中心，广东省妇幼保健院，广东 广州 511443; Department of Clinical Laboratory，Women and Children’s Hospital，Southern University of Science and Technology，Guangdong Women and Children Hospital，Guangzhou 511443，China

**Keywords:** 新生儿败血症, 脂质代谢组学, 脑脊液, 生物标志物, 甘油磷脂代谢, neonatal sepsis, lipid metabolomics, cerebrospinal fluid, biomarker, glycerophospholipid metabolism

## Abstract

新生儿败血症是导致新生儿发病和死亡的主要原因，但目前缺乏敏感、特异的早期生物标志物，尤其是关于脑脊液（CSF）脂质代谢的系统研究仍较有限。本研究纳入17例血培养阳性的败血症新生儿及其同期阴性对照，采用液相色谱-质谱联用技术对其血清与CSF样本进行靶向脂质组学分析。首先通过单变量和多变量分析筛选差异代谢物，然后进行通路富集分析。进一步结合Boruta算法与受试者工作特征（ROC）曲线分析，筛选并评估潜在诊断标志物的效能。结果显示，败血症组血清中除甘油三酯（TAG）和二酰基甘油（DAG）外，其余脂质种类含量均显著低于对照组（*P*<0.05）；CSF中磷脂酰乙醇胺（PE）、神经酰胺（Cer）和溶血磷脂酰乙醇胺（LPE）水平均明显下降（*P*<0.05）。差异分析共识别出血清中107种、CSF中34种显著下调的脂质代谢物，均未发现上调脂质。通路分析提示甘油磷脂代谢在两类体液中均显著富集。血清与CSF中共有13种差异脂质代谢物，其中LPE（18：2）、ePE（36：4）和Cer（d18：1/25：0）在两种体液中的浓度呈显著正相关（Pearson *r*=0.369~0.382，*P*<0.05）。Boruta算法识别出血清中LPC（28：1）、LPE（18：2）与ePE（36：4）3种潜在标志物，曲线下面积（AUC）分别为0.96、0.94和0.94；CSF中Cer（d18：1/26：0）、Cer（d18：1/25：0）和Cer（d18：1/24：1）的AUC为0.89、0.91和0.80，表现出良好的诊断性能。本研究系统揭示了新生儿败血症中血清与CSF脂质代谢的紊乱，尤其甘油磷脂通路在两种体液中均表现出一致性异常，提示中枢与外周代谢存在协同失衡。此外，血清脂质标志物具备良好的早期筛查潜力，CSF脂质变化则提示中枢神经系统在败血症早期可能已受累，具有神经损伤预警价值，该研究为新生儿败血症的精准诊断与发病机制研究提供了新视角。

## Abstract (en)

Neonatal sepsis remains a leading cause of morbidity and mortality among newborns worldwide. Despite advances in neonatal care， early diagnosis of sepsis remains challenging due to the lack of sensitive and specific biomarkers. While serum-based indicators have been widely studied， lipid metabolism in cerebrospinal fluid （CSF） remains relatively underexplored， limiting our understanding of central nervous system involvement （CNS） in the early stages of neonatal sepsis. This study aimed to systematically investigate lipid metabolic alterations in both serum and CSF samples from neonates with confirmed sepsis and to identify potential lipid biomarkers for early diagnosis. Seventeen neonates with blood culture-positive sepsis and seventeen controls with negative blood culture results were enrolled from the Neonatal Intensive Care Unit of Guangdong Women and Children Hospital （Women and Children’s Hospital， Southern University of Science and Technology） between February 2020 and August 2023. Paired serum and CSF samples were collected and analyzed using targeted lipidomics based on liquid chromatography-tandem mass spectrometry （LC-MS/MS）. Univariate analyses， including Student’s *t*-tests and Mann-Whitney U tests， were applied to identify statistically significant differences in lipid levels between groups. Multivariate analyses， including principal component analysis （PCA） and orthogonal partial least squares discriminant analysis （OPLS-DA）， were employed to further evaluate group separation and identify discriminatory lipid species. Pathway enrichment analysis was performed using the Kyoto Encyclopedia of Genes and Genomes （KEGG） database， and candidate biomarkers were selected using the Boruta feature selection algorithm and evaluated for diagnostic performance using receiver operating characteristic （ROC） curve analysis. A total of 322 lipid metabolites were identified in serum， with cholesteryl esters （CE）， triacylglycerols （TAG）， and phosphatidylcholines （PC） being the most abundant lipid classes. In the sepsis group， levels of nearly all lipid subclasses were significantly decreased compared to controls （*P*<0.05）， except for TAG and diacylglycerols （DAG）， which were not significantly altered. In CSF， 300 lipid species were detected， dominated by CE， PC， and phosphatidylethanolamines （PE）. Significantly reduced levels of PE， ceramides （Cer）， and lyso phosphatidylethanolamines （LPE） were observed in septic neonates （*P*<0.05）. PCA plots demonstrated tight clustering of quality control （QC） samples， indicating high analytical reproducibility and stable instrument performance. In serum， PCA accounted for 66.1% of total variance， showing preliminary group separation that was further confirmed by OPLS-DA （*R*²*Y*=0.601， *Q*²*Y*=0.271）， which identified 107 significantly downregulated lipid metabolites. Similarly， CSF PCA explained 75.7% of the variance， and OPLS-DA （*R*²*Y*=0.579， *Q*²*Y*=0.368） revealed 34 significantly downregulated lipid metabolites. Pathway enrichment analysis （FDR-*P*<0.05， pathway impact>0.10） showed that glycerophospholipid metabolism was the most significantly enriched pathway in both serum and CSF， followed by ether lipid and sphingolipid metabolism in serum. Key shared metabolites included PE（42：9）， PC（38：0）， LPC（22：6）， and LPE（22：6）， while PS（40：6） and PI（40：4） were specific to serum. Notably， thirteen differential lipid species were consistently identified in both serum and CSF， among which LPE（18：2）， ePE（36：4）， and Cer（d18：1/25：0） exhibited significant positive correlations between the two fluids （Pearson *r*=0.369-0.382， *P*<0.05）， suggesting potential trans-barrier lipid communication or shared regulatory mechanisms. Boruta-based machine learning analysis identified LPC（28：1）， LPE（18：2） and ePE（36：4） in serum as candidate biomarkers. These exhibited excellent diagnostic performance， with area under the curve （AUC） values of 0.96， 0.94， and 0.94， respectively， sensitivities ranging from 82.4% to 88.2%， and specificities from 94.1% to 100%. In CSF， Cer（d18：1/26：0）， Cer（d18：1/25：0）， and Cer（d18：1/24：1） were identified as high-importance variables. These demonstrated diagnostic AUCs of 0.89， 0.91， and 0.80， with sensitivities between 88.2% and 100% and specificities ranging from 64.7% to 70.6%. In summary， this study provides the first integrated lipidomic profiling of serum and CSF in neonatal sepsis， highlighting a consistent disruption in lipid metabolism， particularly within the glycerophospholipid pathway. Serum lipid biomarkers show promise as non-invasive early screening tools， while CSF lipid alterations offer valuable insights into CNS involvement and potential early neuroinflammatory responses. These findings support the potential of lipid-based biomarkers in improving the precision and timeliness of neonatal sepsis diagnosis. Nevertheless， the relatively small sample size and single-center design may limit the generalizability of the results. Future multicenter studies with larger cohorts are warranted to validate these findings and support clinical translation into neonatal care.

新生儿败血症是一种由病原体感染引起的严重全身性炎症反应，因新生儿免疫系统尚未完全发育，易导致感染进展迅速。全球每年约有130万新生儿受到影响，死亡率高达17.6%^ ［1］^。我国的一项区域性调查显示，其发病率为每1 000活产儿25.6例^［2］^。若不能及时识别和治疗，易导致脑膜炎、弥散性血管内凝血等并发症，影响神经发育^［3］^。尽管血培养为诊断金标准，但存在灵敏度低、出报告慢等局限^［4］^，常用炎症指标如C反应蛋白（CRP）、降钙素原（PCT）等也存在敏感性不足的问题^［5］^，难以满足新生儿败血症早期、精准诊断的临床需求。因此，亟需开发新的高敏感性、高特异性的生物标志物以实现新生儿败血症的早期识别和干预。

近年来研究发现，新生儿败血症存在显著的脂质代谢紊乱，脂质分子在免疫调节和炎症应答中发挥关键作用^［6，7］^。血清脂质如溶血磷脂酰胆碱（lyso phosphatidylcholines，LPC）、鞘脂（sphingolipids，SM）、神经酰胺（ceramides，Cer）等与新生儿败血症的感染程度及预后密切相关，部分脂质比值甚至可预测其死亡风险^［7］^。然而，目前研究多集中于血清，脑脊液（cerebrospinal fluid，CSF）中脂质变化的研究仍较有限。败血症引发的全身性炎症可影响中枢神经系统，通过血脑屏障改变、神经元损伤及神经炎症，导致CSF中脂质代谢谱的异常^［8，9］^，其微小变化或可成为中枢神经系统早期受累的敏感标志物。

血清代谢组学已在新生儿败血症标志物研究中取得初步进展，但稳定性和可重复性仍有限，尚难满足临床需求。相比之下，CSF代谢组更贴近中枢代谢状态，对反映脑部炎症与损伤具有独特优势。尽管采集具有一定侵袭性，其在中枢早期受累识别方面仍具有潜力。本研究联合分析败血症患儿的血清与CSF脂质组特征，旨在筛选具有诊断价值的脂质标志物，并探索其与中枢损伤的潜在关联，为精准诊断提供新思路。

## 1 实验部分

### 1.1 仪器与试剂

ACQUITY UPLC-Xevo TQ-S LC-MS/MS系统（Waters，美国），Allegra X-15R离心机（Beckman Coulter，美国），MSC-100涡旋混匀器（杭州奥盛有限公司，中国）。

质谱纯乙腈、异丙醇以及色谱纯醋酸铵均购自Thermo-Fisher Scientific（美国）。实验用超纯水由Millipore Reference 超纯水系统（美国）制备。同位素内标d7-磷脂酰胆碱（phosphatidylcholine，PC，15：0/18：1）、d7-磷脂酰乙醇胺（phosphatidylethanolamine，PE，15：0/18：1）、d7-磷脂酰丝氨酸（phosphatidylserine，PS，15：0/18：1）、d7-磷脂酰肌醇（phosphatidylinositol，PI，15：0/18：1）、d7-LPC（18：1）、d7-溶血磷脂酰乙醇胺（lyso phosphatidylethanolamine，LPE，18：1）、d7-胆固醇酯（cholesteryl ester，CE，18：1）、d7-甘油二酯（diacylglycerol，DAG，15：0/18：1）、d7-甘油三酯（triacylglycerol，TAG 15：0/18：1/15：0）、d9-SM（d18：1/16：0）与d7-Cer（d18：1/16：0）均购自Avanti Polar Lipids（美国）。

### 1.2 人群资料

所有研究对象均来自广东省妇幼保健院（南方科技大学附属妇女儿童医院）新生儿重症监护病房，于2020年2月至2023年8月期间纳入。新生儿败血症的诊断依据《新生儿败血症诊断及治疗专家共识（2019年版）》，包括：（1）发热、低体温、呼吸困难、黄疸、呕吐、腹泻等非特异性症状；（2）血培养阳性；（3）实验室指标异常，如白细胞或中性粒细胞升高、CRP升高、血小板减少；（4）辅助检查提示感染证据（如胸片、尿液或CSF检查）。两组研究对象均排除以下情况：（1）日龄>28天；（2）合并脑膜炎（包括CSF细胞数升高、蛋白升高、葡萄糖下降、液体混浊、培养阳性或影像学提示脑膜受累）；（3）样本不足或溶血严重；（4）遗传代谢病或严重先天畸形；（5）病历资料不全。最终纳入血培养阳性、未合并脑膜炎的新生儿败血症病例17例；同期住院、血培养阴性、无败血症诊断的新生儿17例作为对照。两组患儿CSF培养结果均为阴性。本研究方案已获得广东省妇幼保健院伦理审查委员会批准（批准编号：No. 202001102）。

### 1.3 样本采集与资料收集

新生儿样本均由经过专业培训的医护人员在严格的无菌条件下采集，采集前已获得父母或法定监护人的知情同意。血液样本由护士使用促凝真空采血管采集静脉血，在3 000 r/min条件下离心，分离获得血清用于后续检测分析。CSF通过腰椎穿刺采集后，分装至3支无菌试管中，分别用于常规检查、生化分析和细菌培养。所有样本在采集后由检验医生立即送达实验室进行检测，剩余部分经分装后储存在-80 ℃冰箱中，备用于后续研究。本研究所用样本均来源于临床常规检查中剩余的血液和CSF，未增加额外的有创操作。新生儿的基本信息（如性别、出生体重、胎龄、出生年龄）以及入院诊断、出院诊断和感染病原种类等临床资料，均通过医院电子病历系统提取，确保数据完整性与准确性。

### 1.4 样本处理

血清或CSF样本在冰浴条件下解冻后，取10 µL样品加入至96孔板中，加入300 µL含内标的5 mmol/L乙酸铵的甲醇溶液，充分涡旋混合20 min。随后以4 000 r/min离心20 min，取20 µL上清液转移至新的96孔板中，加入80 µL含5 mmol/L乙酸铵的甲醇溶液，再次涡旋混合10 min后用于进样分析。

本研究采用混合样本作为质量控制（quality control，QC）措施，以评估仪器稳定性与数据一致性。在样本前处理之前，分别从每例血清和CSF样本中各取10 μL，转移至1.5 mL离心管中，涡旋混匀10 min以构建血清与CSF混合样本池，作为相应体液类型的QC样本来源。随后，QC样本按照与研究样本一致的流程进行前处理。在样本分析过程中，QC样本于每批次检测的起始和结束各插入1次，同时每检测17个实际样本插入1次。最终，在血清与CSF分析过程中分别测定QC样本共3次。

### 1.5 分析条件

色谱分离采用ACQUITY UPLC BEH C18柱（100 mm×2.1 mm， 1.7 µm），柱温40 ℃，样品管理器温度10 ℃。流动相A为乙腈-水（60∶40，含5 mmol/L甲酸铵和0.1%甲酸），B为异丙醇-乙腈（90∶10，含5 mmol/L甲酸铵和0.1%甲酸），梯度洗脱程序：0~0.5 min，60%B；0.5~3 min，60%B~80%B；3~7 min，80%B~100%B；7~9 min，100%B；9~9.5 min，100%B~60%B；9.5~11 min，60%B。流速0.30 mL/min，进样体积2.0 µL。质谱采用ESI正离子模式，毛细管电压3.0 kV，离子源温度150 ℃，脱溶剂温度550 ℃，脱溶剂气流速1 000 L/h，碰撞气流速0.13 L/h。为避免仪器波动或操作因素所带来的误差，对样本进行随机进样分析。在实验过程中，每批次样本分析前后均设置混合质控样本，以监测分析过程中的数据质量。仪器生成的原始数据文件采用MassLynx软件（V4.1，Waters， USA）进行初步处理，然后通过TMBQ软件（V1.0，Metabo-Profile，上海）对提取离子色谱峰进行提取、积分和定量。本研究共检测分析375种脂质代谢物，涵盖CE、TAG、PC、PE、LPC、DAG、SM、Cer、PI、PS、LPE、神经酰胺磷酸乙醇胺（ethanolamine phosphoryl ceramide，CerPE）、磷脂酸（phosphatidic acid，PA）、醚磷脂酰乙醇胺（ether phosphatidylethanolamine，ePE）与醚磷脂酰丝氨酸（ether phosphatidylserine，ePS）类。各类脂质代谢物均依据其结构特征进行脂质类别分类，并采用结构相似的同位素内标，通过待测物与内标的峰面积比值实现定量分析。

### 1.6 数据分析

对研究人群的基本特征和实验室指标进行描述性统计分析。符合正态分布的连续变量以均数±标准差（standard deviation，SD）表示，组间比较采用独立样本*t*检验；不符合正态分布的变量以中位数（四分位数）表示，采用Mann-Whitney U检验。分类变量以频数和百分比表示，组间比较使用Pearson卡方检验或Fisher精确检验。

脂质组学数据经对数转换后，进行单变量分析，包括*t*检验和差异倍数（fold change，FC）分析，所得结果通过假发现率（false discovery rate，FDR）方法进行多重比较校正。差异代谢物通过火山图等方式进行可视化展示。数据经*Z*-score标准化后，采用主成分分析（principal component analysis，PCA）和正交偏最小二乘判别分析（orthogonal partial least squares discriminant analysis，OPLS-DA）进行多变量建模，并计算变量投影重要性（variable importance in projection，VIP）值以评估各代谢物的判别能力。差异代谢物的筛选基于单变量与多变量分析，标准为|log₂ FC|>1、FDR-*P*<0.05且VIP>1。代谢通路分析基于筛选出的显著差异代谢物，通过KEGG数据库进行富集分析与通路映射，并使用气泡图展示显著通路特征。潜在生物标志物的筛选采用基于随机森林算法的Boruta特征选择方法，结合与影子特征比较识别分类能力稳定的关键代谢物。最终，应用受试者工作特征曲线（receiver operating characteristic curve，ROC）评估其诊断性能，并计算曲线下面积（area under the curve，AUC）评价模型判别效能。上述分析通过MetaboAnalyst平台（https：//www.metaboanalyst.ca/）及R语言4.2.2版本完成。

## 2 结果与讨论

### 2.1 研究对象的一般资料

表1结果显示，新生儿败血症组的出生身长与出生孕周显著低于对照组（39.50 vs. 49.00 cm，*P*=0.004；33.49 vs. 36.85周，*P*=0.015），而胎儿性别、日龄和出生体重不存在显著差异（*P*>0.05）。实验室指标方面，PCT、CRP、白细胞（WBC）、中性粒细胞（NEUT）与中性粒细胞百分比（NEUT%）在两组间均无显著差异（*P*>0.05）。本研究中，大肠埃希菌为检出最多的致病菌（6株，33.3%），其次为肺炎克雷伯菌（3株，16.7%）与金黄色葡萄球菌（2株，11.1%）。其余包括粪肠球菌、屎肠球菌、无乳链球菌、嗜麦芽黄单胞菌、溶血葡萄球菌、裴氏棒状杆菌及咽峡炎链球菌，各检出1株。其中1例患儿同时感染粪肠球菌与肺炎克雷伯菌，属混合感染情况。

**表1:** 研究人群基本特征与病原菌检出情况

Characteristics	Case （*n*=17）	Control （*n*=17）	*P*-value
Male infant	13 （76.5%）	10 （58.8%）	0.271
Age/day	12.24±10.50	10.00±7.86	0.501
Birth weight/kg	1.65 （1.10-2.97）	2.75 （1.93-3.36）	0.079
Birth length/cm	39.50 （36.5-41.75）	49.00 （44.50-50.25）	0.004^*^
Gestational age/week	33.49±4.37	36.85±3.06	0.015^*^
PCT/（ng/mL）	0.35 （0.18-1.47）	0.56 （0.30-1.45）	0.527
CRP/（mg/L）	2.32 （0.52-7.45）	4.34 （0.91-34.50）	0.433
WBC/（10^9^/L）	13.04 （8.22-16.55）	11.05 （8.04-12.96）	0.260
NEUT/（10^9^/L）	7.30 （4.17-13.83）	5.27 （3.42-7.84）	0.217
NEUT%/%	60.90 （41.03-78.03）	58.00 （38.35-64.95）	0.204
Types of infectious agents			
*Escherichia coli*	6 （33.3%）	/	/
*Klebsiella pneumoniae*	3 （16.7%）	/	/
*Staphylococcus aureus*	2 （11.1%）	/	/
Other pathogens	7 （39.2%）	/	/

Data are presented as *n*（%）， mean±SD， or median （25^th^-75^th^ percentile）. PCT： procalcitonin； CRP： C-reactive protein； WBC： white blood cell； NEUT： neutrophil； Other pathogens include *Enterococcus faecium*， *Enterococcus faecalis*， *Streptococcus agalactiae*， *Sphingomonas maltophilia*， *Staphylococcus haemolyticus*， *Corynebacterium pyogenes*， and *Streptococcus anginosus*. One case involved a mixed infection with *Enterococcus faecium* and *Klebsiella pneumoniae*. *P<0.05.

### 2.2 血清与CSF脂质代谢物分布特征

在血清样本中共鉴定出322种脂质代谢物。如图1a所示，CE、TAG和PC为含量最丰富的3类脂质，在对照组中分别占比70.3%、20.2%和3.2%，在病例组中则分别为61.8%、26.9%和4.3%。除TAG和DAG外，病例组中其他脂质种类的水平均显著低于对照组（*P*<0.05）。在CSF样本中，共检出300种脂质代谢物。如图1b所示，CE、PC和PE构成了CSF脂质的主要成分，在对照组中分别占比62.0%、14.4%和15.3%，在病例组中则分别为67.9%、18.8%和4.7%。与对照组相比，病例组CSF中PE、Cer与LPE 水平显著降低（*P*<0.05）。本研究结果表明，新生儿败血症存在明显的脂质代谢紊乱，表现为血清和CSF中多种脂质成分显著下调，提示脂质代谢可能在新生儿败血症的发病过程中发挥重要作用。

**图1:**
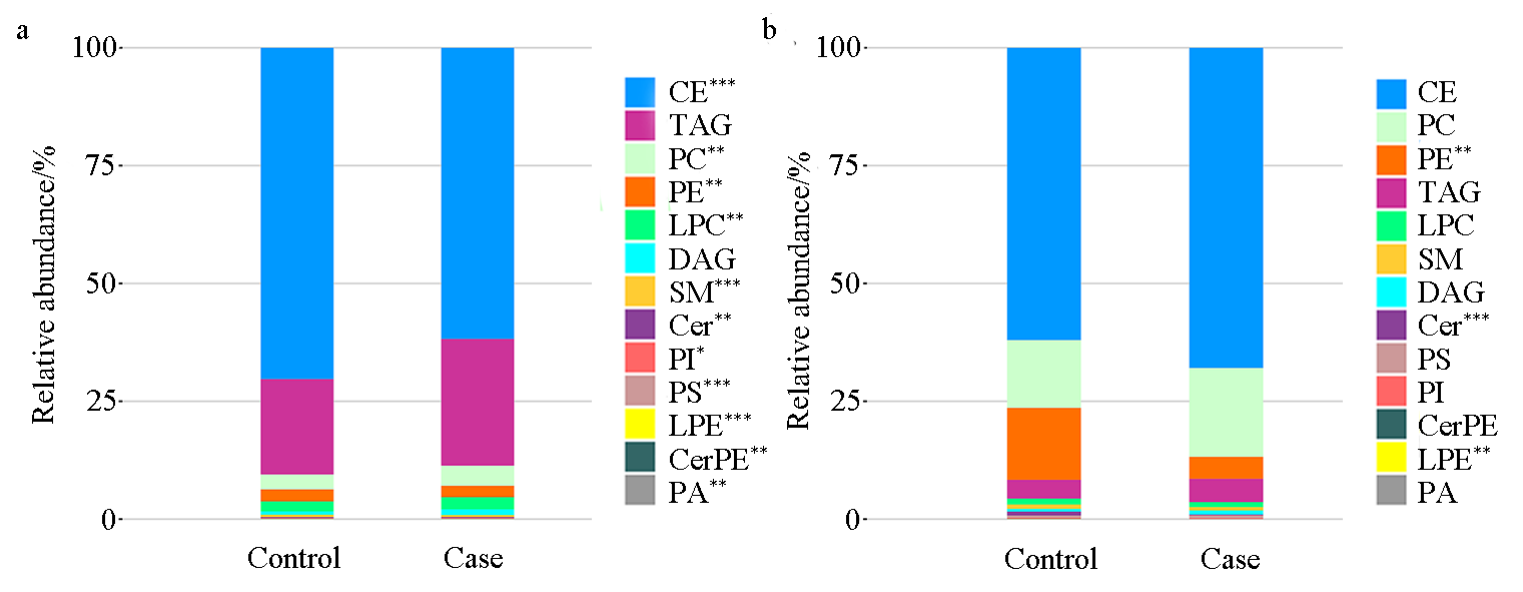
（a）血清和（b）CSF样本脂质代谢物的分布情况

### 2.3 血清与CSF脂质组学的差异代谢物分析

在血清与CSF样本的PCA图中，所有QC样本在两组中均呈紧密聚集分布，表明检测过程中仪器运行稳定、数据重复性良好。血清样本的PCA结果（图2a）显示，前两个主成分共解释66.1%的总方差，提示两组在整体脂质谱上存在初步的区分趋势。进一步采用OPLS-DA构建判别模型（图2c），显著提升了组间的区分度。通过1 000次置换检验评估模型的可靠性，结果显示模型拟合度（*R*²*Y*=0.601）与预测能力（*Q*²*Y*=0.271）未出现过拟合（图2e）。依据VIP值和统计学检验，共筛选出107种在病例组中显著下调的差异脂质代谢物（图2h）。在CSF样本中，PCA前两个主成分累计解释75.7%的总方差（图2b），但组间分离不明显。OPLS-DA模型（图2d）第一预测主成分（P1）和第一正交主成分（O1）分别解释16.5%和53.4%的总方差。置换检验显示模型具有较好的拟合（*R*²*Y*=0.579）和预测能力（*Q*²*Y*=0.368，图2f），最终共鉴定出34种在病例组中显著下调的差异脂质代谢物（图2g）。

**图2:**
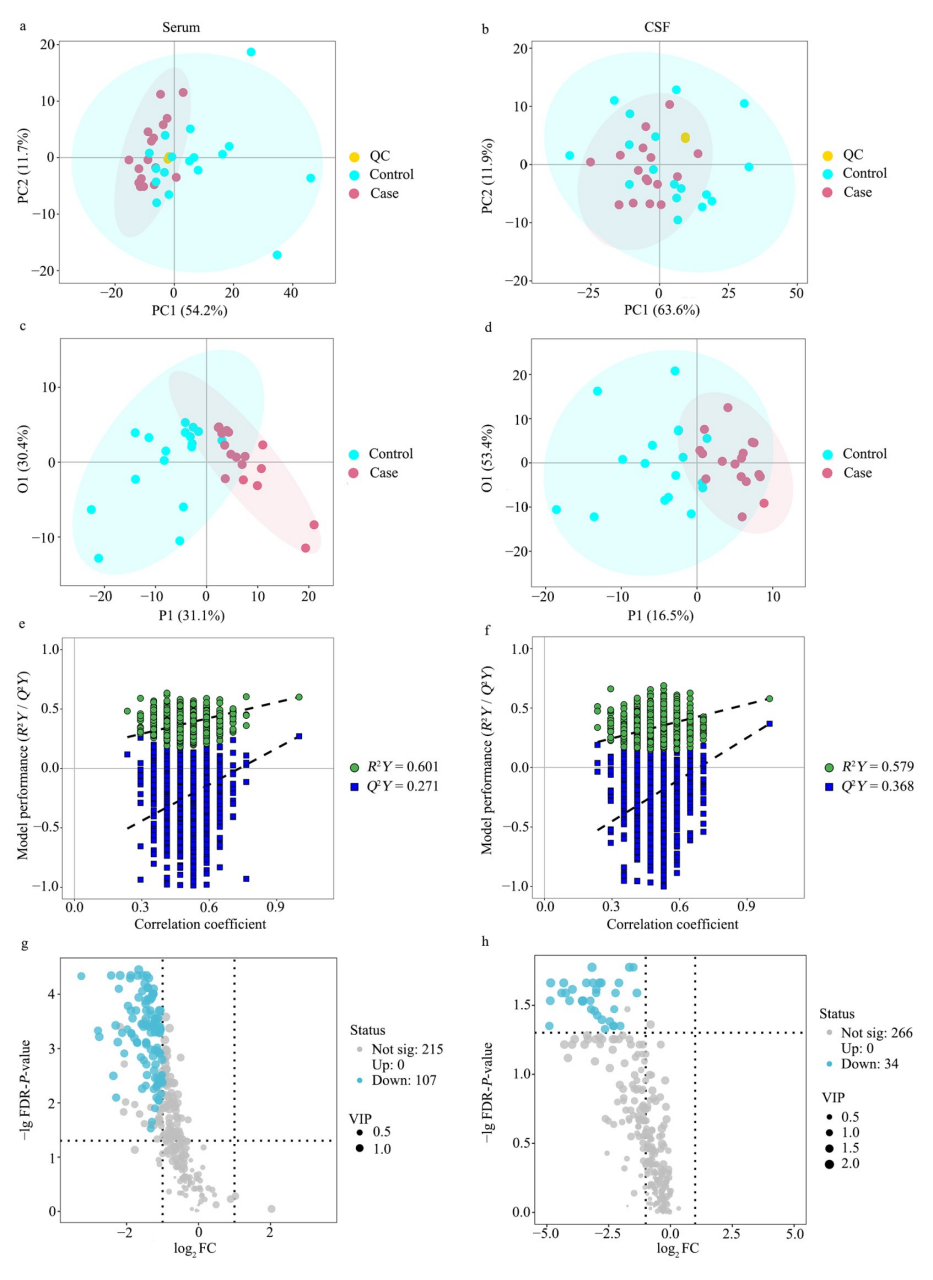
血清与CSF脂质组学的差异代谢物分析

已有研究表明，败血症会导致关键脂质如PE、LPE、LPC及SM下调，这些变化与免疫激活、炎症加剧、血脑屏障破坏及器官功能障碍密切相关^［6］^。例如，Wang等^［10］^通过代谢组学发现，败血症患儿血清中PE（16：0/18：2）、PA（8：0/14：0）及SM（d18：0/16：1）均显著下调；而另一项研究亦指出^［11］^，在脓毒症患者中，多数脂质分子（包括PC、LPC、LPE及部分PE）表达水平显著降低。值得注意的是，本研究在新生儿CSF中也观察到了类似的脂质下调现象，提示中枢脂质代谢在败血症早期已受累，可能与神经炎症或细胞损伤密切相关^［8］^。研究表明，磷脂、肉碱和色氨酸等脂质相关代谢物在细菌性和病毒性脑部感染中均表现出特征性变化，提示CSF脂质谱的变化可作为传统CSF指标的补充，有望用于早期识别中枢神经系统受累^［12］^。

### 2.4 差异脂质分子的代谢通路分析

本研究将血清中107种以及CSF中34种差异代谢物进行通路富集分析。根据FDR-*P*<0.05与pathway impact>0.1的判断标准，血清样本中显著富集的通路包括甘油磷脂代谢（glycerophospholipid metabolism）、醚脂代谢（ether lipid metabolism）和鞘脂代谢（sphingolipid metabolism），其中甘油磷脂代谢通路富集度最高（pathway impact=0.30）（图3a）。同样，CSF分析结果亦显示甘油磷脂代谢是主要富集通路（pathway impact=0.23）（图3b）。图3c展示了甘油磷脂代谢通路的代谢网络结构，其中PE（42：9）（ID： C00350）、PC（38：0）（ID： C00157）、LPC（22：6）（ID： C04230）和LPE（22：6）（ID： C04438）为血清与CSF样本中的共同差异代谢物，而PS（40：6）（ID： C02737）和PI（40：4）（ID： C01194）仅在血清中有差异。

**图3:**
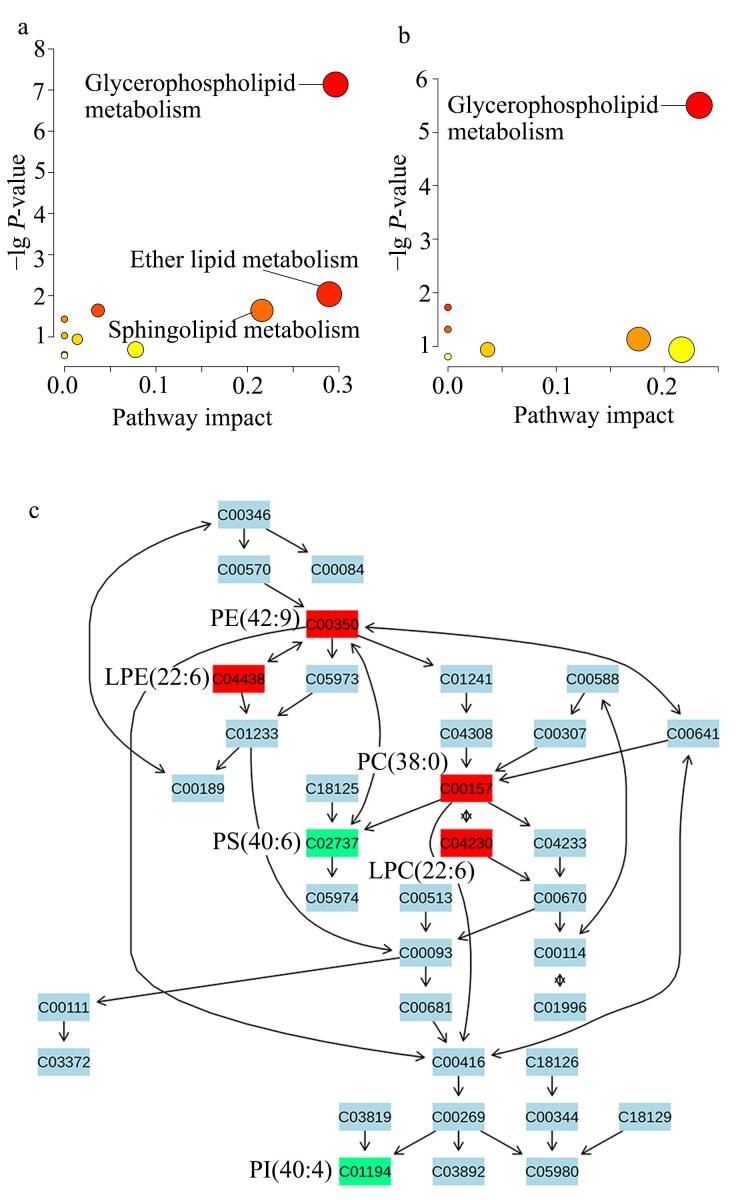
（a）血清和（b）CSF样本中脂质代谢物的通路富集分析与（c）甘油磷脂代谢通路

本研究发现甘油磷脂代谢通路在新生儿败血症中显著紊乱，多个关键分子在该通路中具有较高的中心性，提示其在疾病进程中可能发挥重要作用。甘油磷脂是细胞膜的主要成分，广泛参与膜结构维持、信号转导和免疫调控^［13］^。研究中血清与CSF中PC、PE、LPC等甘油磷脂显著下调，反映出系统性与中枢性的脂质代谢异常，可能影响细胞膜的稳定性并加剧炎症与器官损伤。已有研究指出，PC代谢产物可作为炎症介质，甘油磷脂广泛下调是败血症的代谢特征之一^［11，14］^。本研究证实血清与CSF中甘油磷脂通路存在协同紊乱，提示中枢代谢应激反应可能早期发生。Chang等^［15］^亦在成人脓毒症中发现LPE、PE、PC为关键代谢物。尽管新生儿CSF脂质研究较少，已有动物和临床数据提示其代谢变化与神经损伤相关^［8，16］^。本研究结果表明，即使未合并脑膜炎，新生儿CSF中甘油磷脂已显著下调，提示其可作为中枢受累的早期代谢标志，值得深入探索其机制与诊断价值。

### 2.5 血清与CSF脂质代谢物的相关性分析

将血清中检出的107种差异代谢物与CSF中检出的34种差异代谢物进行韦恩图分析，发现13种共同的差异代谢物，包括PE（34：4）、PE（34：2）、PC（38：0）、LPE（18：2）、LPE（18：1）、LPE（16：0）、LPC（26：0）、ePE（38：4）、ePE（36：4）、Cer（d18：1/25：0）、Cer（d18：1/24：0）、Cer（d18：1/23：0）与Cer（d18：0/24：0）。 进一步分析发现（图4），LPE（18：2）、ePE（36：4） 和Cer（d18：1/25：0）在两种体液中的浓度呈显著正相关（Pearson *r*=0.369~0.382，*P*<0.05），提示这些脂质在败血症状态下可能受相似代谢或炎症调控机制影响。新生儿血脑屏障尚未发育完全，其结构和功能均不成熟，通透性显著高于成年人^［17］^。在全身炎症状态下，炎症因子如TNF-α、IL-6等可进一步破坏血脑屏障的结构与功能，使得部分脂质分子能够在血清与CSF之间发生转运，从而形成代谢协同紊乱^［17］^。这一结果具有重要临床意义，表明部分血清脂质代谢物在一定程度上可反映CSF中脂质代谢紊乱的程度，或可作为中枢系统受累的替代指标，用于提示新生儿败血症相关神经功能损害的风险。

**图4:**
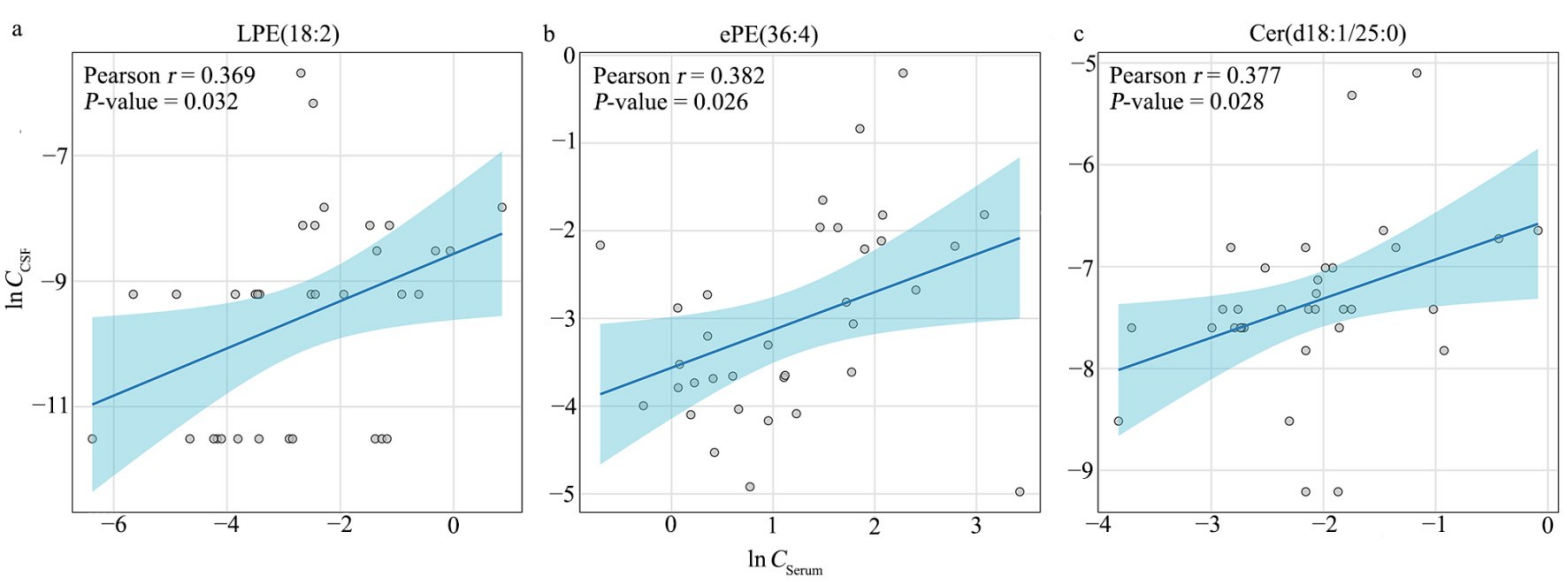
脂质代谢物在血清与CSF中的相关性分析

### 2.6 基于Boruta特征选择的诊断实验结果

本研究通过Boruta特征选择，从血清中107种差异脂质中筛选出35个具有稳定判别能力的变量，LPC（28：1）、LPE（18：2）和ePE（36：4）得分最高，作为潜在诊断指标用于ROC分析。结果（图5a~c）显示，LPC（28：1）、LPE（18：2）和ePE（36：4）的AUC分别为0.96、0.94和0.94，敏感度为82.4%~88.2%，特异度为94.1%~100%，表现出良好的诊断性能。本研究筛选出的3种血清脂质代谢物在新生儿败血症中表现出优异的诊断性能，AUC均≥0.94，特异度最高达100%。相比传统指标中等偏下的灵敏度，如PCT（81.25%）、SAA（75.00%）及CRP（71.88%）^［18］^，本研究脂质标志物更具敏感性和特异性，且机制明确、临床转化潜力大。另有研究提示，早发败血症患儿血清中PCT和CRP的灵敏度仅为66.67%和33.33%^［19］^，而血清miR-15b和miR-378a的灵敏度分别为76%与60%，虽具有一定诊断价值，但检测过程复杂，尚难以实现临床快速应用^［20］^。相较之下，脂质代谢物在诊断效能、机制支撑和应用可行性方面均表现出明显优势。

**图5:**
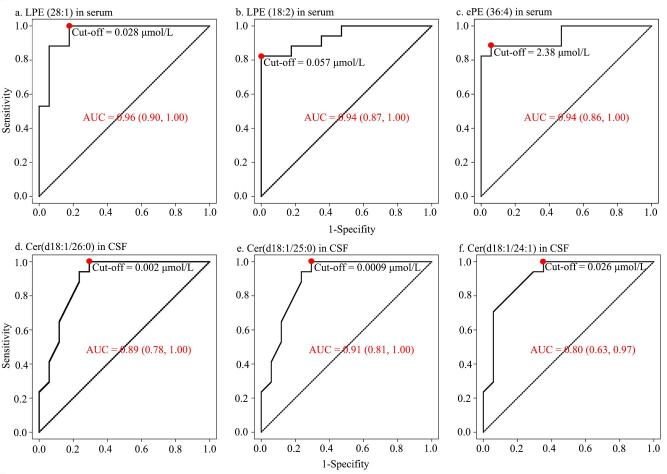
血清与CSF脂质代谢物的ROC曲线

LPC（28：1）与LPE（18：2）是甘油磷脂代谢通路中的关键中间产物，广泛参与炎症和细胞凋亡。LPC可通过G蛋白偶联受体活化巨噬细胞，诱导线粒体损伤、细胞死亡，还能增强吞噬作用、抑制中性粒细胞陷阱释放、降低死亡率^［21，22］^，并通过激活NLRP3炎性小体加重炎症^［23］^。其在新生儿败血症中显著下降，与28天死亡率相关，具有诊断和预后价值^［24， 25］^。LPE同样具有抗炎、调控白细胞渗出和因子释放的功能^［26］^，其衍生物熊去氧胆酸-LPE在肝损伤模型中抗炎作用更强^［27］^，并对部分革兰氏阳性菌具有抗菌作用^［28］^。ePE在感染模型中亦显著下降，可能通过PLA2介导分解、膜脂氧化和线粒体损伤参与脂质重构^［29］^，但在新生儿败血症中的作用尚需进一步研究。

本研究在34种CSF差异脂质中筛选出7个具有稳定判别能力的变量，其中Cer（d18：1/26：0）、Cer（d18：1/25：0）和Cer（d18：1/24：1）得分最高，作为潜在诊断指标用于ROC分析。结果显示（图5d~f），三者AUC分别为0.89、0.91和0.80，敏感度为88.2%~100%，特异度为64.7%~70.6%。本研究发现的3种CSF脂质在新生儿败血症中具有较好的诊断效能，尤其是Cer（d18：1/26：0）与Cer（d18：1/25：0）的敏感度达100%，在排除败血症方面具有较高价值。尽管患儿未合并脑膜炎，CSF中Cer水平仍显著下降，提示中枢或已发生脂质代谢紊乱，可能与早期脑部炎症或神经损伤相关。相比血清标志物，这些Cer类物质虽诊断性能稍逊，但在提示中枢受损方面具有独特意义。已有研究指出，Cer代谢紊乱在多种神经系统疾病如阿尔茨海默病、抑郁症和多发性硬化中发挥关键作用。在成人脓毒症中，Cer水平普遍升高，且与脑损伤及病死率相关^［30，31］^。但本研究中，新生儿败血症患儿CSF中的Cer类物质均显著下调，反映其脂质代谢应答与成年人存在明显差异。一方面，新生儿脑组织中神经元尚处于发育阶段，Cer的合成能力有限；另一方面，新生儿免疫系统以Th2型为主，促炎因子反应较弱，不足以激活鞘磷脂酶或神经酰胺从头合成途径，反而因能量代谢障碍抑制神经酰胺合成^［32， 33］^。此外，尚未发育完全的血脑屏障通透性增加，也可能导致Cer外渗，使其在CSF中浓度下降^［17］^。因此，Cer类物质在新生儿中的生理功能及其病理变化机制仍需深入研究，成人的抗炎策略未必适用于新生儿，甚至可能干扰其脂质稳态。

## 3 结论

本研究系统揭示了新生儿败血症中血清与CSF脂质代谢的紊乱，尤其甘油磷脂通路在两种体液中均表现出一致性异常，提示中枢与外周代谢存在协同失衡。血清中的LPC（28：1）、LPE（18：2）和ePE（36：4）表现出良好的诊断性能，具有成为无创生物标志物的潜力；CSF中Cer类脂质的变化则提示中枢系统可能在败血症早期即已受累。本研究为新生儿败血症的精准诊断与神经损伤预警提供了新的研究视角和理论支持。然而，本研究样本量较小且为单中心设计，可能限制结果的统计效能和外部泛化能力。未来应在多中心、大样本基础上进一步开展验证研究，以提升脂质标志物的稳定性与临床转化价值。
